# The Anti-Angiogenic Activity of a Cystatin F Homologue from the Buccal Glands of *Lampetra morii*

**DOI:** 10.3390/md16120477

**Published:** 2018-11-29

**Authors:** Mingru Zhu, Bowen Li, Jihong Wang, Rong Xiao

**Affiliations:** School of Life Sciences, Liaoning Normal University, Dalian 116081, China; zhumingru_72@163.com (M.Z.); libw@mail.dlut.edu.cn (B.L.); y.y.200@163.com (J.W.)

**Keywords:** *Lampetra morii*, buccal gland, cystatin F, anti-angiogenesis, cystatin superfamily

## Abstract

Cystatins are a family of cysteine protease inhibitors which are associated with a variety of physiological and pathological processes in vivo. In the present study, the cDNA sequence of a cystatin F homologue called Lm-cystatin F was cloned from the buccal glands of *Lampetra morii*. Although Lm-cystatin F shares a lower homology with cystatin superfamily members, it is also composed of a signal peptide and three highly conserved motifs, including the G in the N-terminal, QXVXG, as well as the PW in the C-terminal of the sequence. After sequence optimization and recombination, the recombinant protein was expressed as a soluble protein in *Escherichia coli* with a molecular weight of 19.85 kDa. Through affinity chromatography and mass spectrometry analysis, the purified protein was identified as a recombinant Lm-cystatin F (rLm-cystatin F). Additionally, rLm-cystatin F could inhibit the activity of papain. Based on MTT assay, rLm-cystatin F inhibited the proliferation of human umbilical vein endothelial cells (HUVECs) dose dependently with an IC_50_ of 5 μM. In vitro studies show that rLm-cystatin F suppressed the adhesion, migration, invasion, and tube formation of HUVECs, suggesting that rLm-cystatin F possesses anti-angiogenic activity, which provides information on the feeding mechanisms of *Lampetra morii* and insights into the application of rLm-cystatin F as a potential drug in the future.

## 1. Introduction

Cystatins are a group of inhibitors, which could suppress the activity of C1 cysteine proteases and play important roles in a variety of physiological process to protect our tissues from inappropriate proteolysis [1,2]. Regarded as inhibitors, cystatins would also cooperate with cathepsins to regulate a series of events, including the maturation of dendritic cells, antigen processing and presentation, phagocytosis, as well as the expression of cytokines [1,3,4,5,6]. At any moment, cystatins are expressed or secreted to control the activity of cathepsin B, L, C, S, H, or other C1 cysteine proteases strictly. Once the dynamic equilibrium between the levels of cystatins and their substrates is destroyed, people might suffer diseases, such as malignant tumor, neurodegenerative diseases, cardiovascular disease, and chronic kidney disease [1,7,8,9,10].

In bloodsucking animals, such as ticks, cystatins are usually expressed in their salivary glands or the midgut, which suggests that cystatins might participate in the feeding process of bloodsuckers [11,12,13,14,15]; while in snakes, the cystatins usually exist in their venom glands, which have been reported to suppress the growth, invasion, and metastasis of B16F10 cells and MHCC97H cells, as well as to inhibit tumor angiogenesis [16,17,18,19]. At present, more cystatins have been identified from venomous insects, snakes, fishes, or mollusks through gene cloning, and transcriptomic and proteomic approaches [20,21,22,23,24]. Regrettably, their biological functions still need further studies.

In our previous study, the protein components of the buccal gland secretion from the fasting and feeding *Lampetra morii* (*L. morii*), which also suck the blood of fishes to survive, were compared through proteomic assays [25]. Among the diverse proteins emerged, a cystatin F homologue (also called as Lm-cystatin F), was identified in the buccal gland secretion of *L. morii* that had been fed on the blood of a catfish for 60 min, which suggests that Lm-cystatin F is closely related to the parasitic mechanisms of the *L. morii* (Figure S1).

To date, cystatin F from the other vertebrates and invertebrates was extensively studied [6,26]. However, little is known about the cystatin F from the buccal glands of *L. morii*, which are one of the most primitive vertebrates still alive. In the present study, a cystatin F homologue from the buccal glands of *L. morii* was cloned, recombined, and expressed. Additionally, its effects on the activity of papain and the endothelial cells (human umbilical vein endothelial cells, HUVECs) were also investigated.

## 2. Results

### 2.1. A Cystatin F Homologue was Identified from the Buccal Glands of L. morii

**Figure 1:**
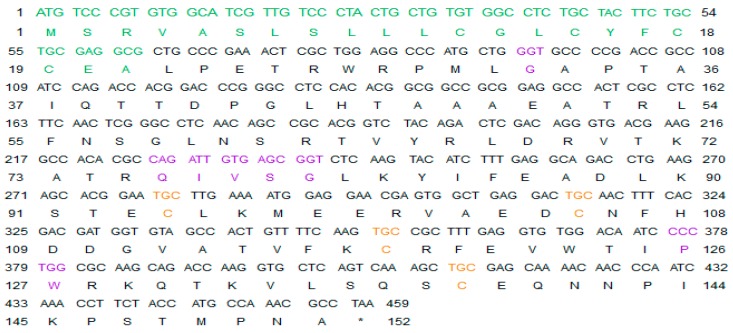
The ORF sequence of Lm-cystatin F and its deduced amino acid sequences. The upper lines show the ORF sequence of Lm-cystatin F, and the lower lines show its deduced amino acid sequence. The sequences are numbered from methionine, and terminated with stop codon. The signal peptide is shown in green; while the three conserved motifs are shown in purple, respectively. Except the cysteines in the signal peptide, the other four cysteines are indicated with orange.

**Figure 2:**
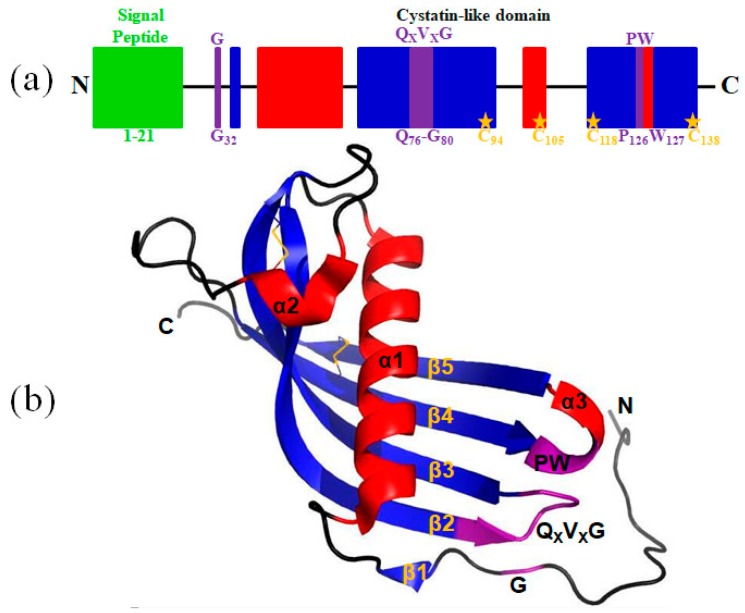
A schematic diagram of Lm-cystatin F and its predicted three-dimensional structure. (**a**) The diagrammatic structure of Lm-cystatin F. The signal peptide and the three conserved motifs are shown in green and purple, respectively. Except the cysteines in the signal peptide, the other four cysteines are labeled with orange. (**b**) The spatial structure of Lm-cystatin F was simulated with the three-dimensional structure of human cystatin F reported in the previous study [27]. The three α helixes and five β sheets are shown with red and blue, respectively. The two disulfide bonds are shown with orange.

As shown in Figure 1, the open reading frame (ORF) sequence of Lm-cystatin F is 459 bp, which encodes 152 amino acids. The predicted molecular weight and theoretical isoelectric point of Lm-cystatin F are 17.1 kDa and 10.31, respectively. Noticeably, the sequence of Lm-cystatin F contains eight rare codons, including four codons for arginines (AGG, AGA, CGA), three for prolines (CCC), and one for leucine (CTA). Based on the analysis on the website (http://www.cbs.dtu.dk/services/SignalP/), the signal peptide sequence of Lm-cystatin F is MSRVASLSLLLCGLCYFCCEA, which indicated that Lm-cystatin F might be secreted extracellularly (Figure 1, green). Similar to the cystatin F from the other species, Lm-cystatin F also contains three highly conserved motifs, which could interact with the cysteine proteases, including the G in the N-terminal, QXVXG, as well as the PW in the C-terminal of the sequence (Figure 1 and Figure 2a). Furthermore, the amino acid sequence of Lm-cystatin F possesses eight cysteines, and four cysteines located at the signal peptide region (Figure 1). The nucleotide sequence of *Lm-cystatin F* has been submitted to the GenBank database (accession number: MG902948). Based on the three-dimensional structure of human cystatin F reported in the previous study, the mimetic structure of Lm-cystatin F was performed and it contains three α helixes, 5 β sheets, two loops (L1 and L2), and two disulfide bonds (Figure 2b) [27].

### 2.2. Sequence Alignment and Phylogenetic Tree

**Figure 3:**
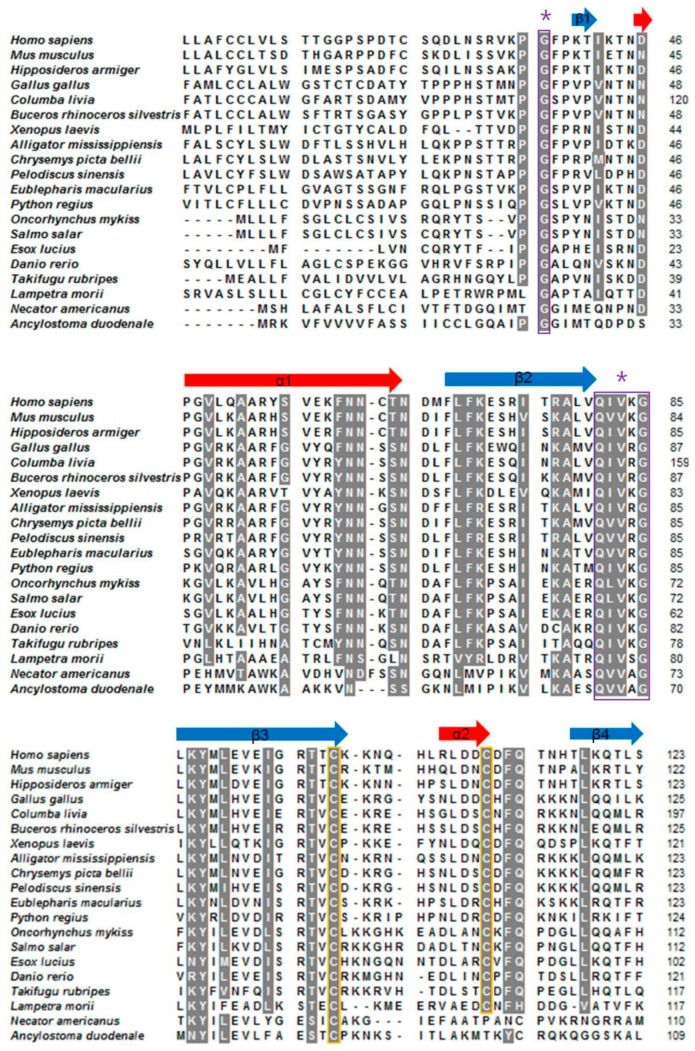

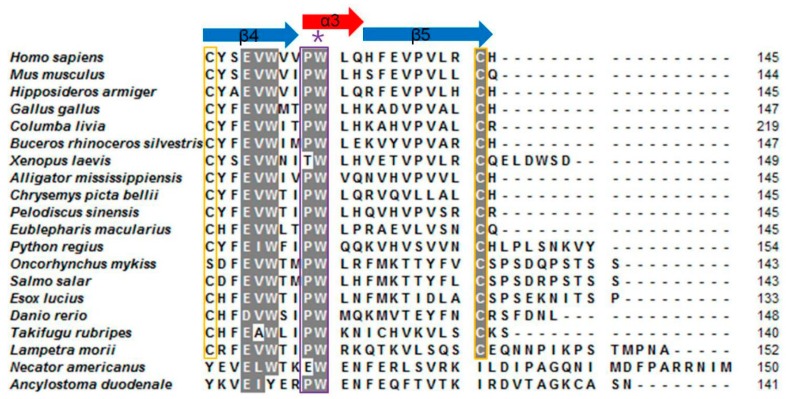
Sequence alignment of 20 cystatin F from the species mentioned previously. Except Lm-cystatin F, the sequences of 19 cystatin F were obtained from the EXPASY database and their accession numbers are listed as followed: *Homo sapiens*, CAD52872.1; *Mus musculus*, NP_034107.2; *Hipposideros armiger*, XP_019488972; *Gallus gallus*, NP_001186323; *Columba livia*, PKK20598.1; *Buceros rhinoceros silvestris*, XP_010137948.1; *Xenopus laevis*, NP_001091281; *Alligator mississippiensis*, KYO18358.1; *Chrysemys picta bellii*, XP_005293586; *Pelodiscus sinensis*, XP_006136954; *Eublepharis macularius*, JAC94872; *Python regius*, JAC94922; *Oncorhynchus mykiss*, XP_021420522.1; *Salmo salar*, NP_001134364.1; *Esox lucius*, NP_001297968.1; *Danio rerio*, NP_001082882; *Takifugu rubripes*, XP_011601516; *Necator americanus*, ETN77353.1; *Ancylostoma duodenale*, KIH58790.1. Additionally, the nucleotide sequence of Lm-cystatin F from *L. morii* was submitted to Genbank (accession number: MG902948). Dashes (-) indicate gaps inserted into the alignment. Asterisks (*) indicate the identical residues. The highly conserved motifs and cysteines are covered with purple and yellow frames, respectively.

**Figure 4:**
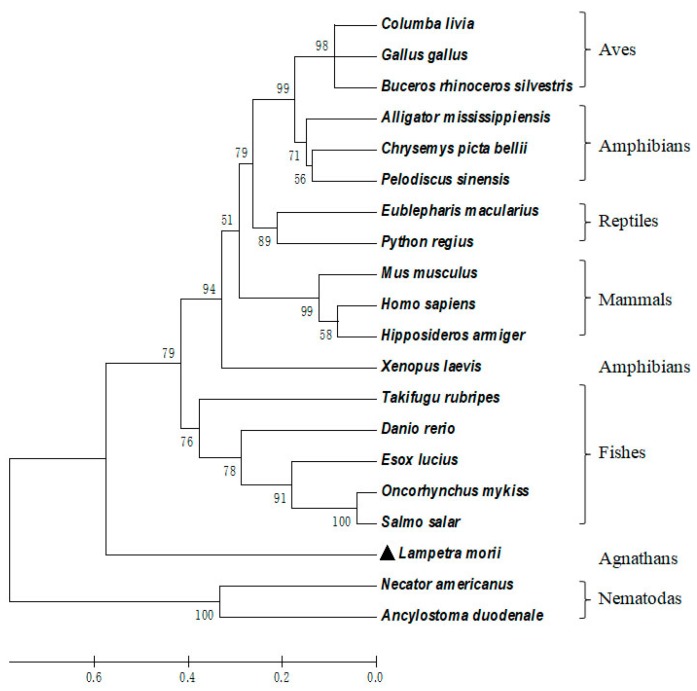
Phylogenetic tree of 20 cystatin F, including Lm-cystatin F. A phylogenetic tree was constructed according to the amino acid sequences of cystatin F listed in Figure 3. The number at each node indicates the percentage of bootstrapping after 1000 replications. The scale bar indicates the average number of amino acid substitutions per site.

As shown in Figure 3, multiple sequence alignment showed that the three motifs of Lm-cystatin F are highly conserved. In addition to the three conserved motifs, the homology between Lm-cystatin F and cystatin F from the other species is not very high. As shown in Table 1, Lm-cystatin F shares 26–38% homology with the cystatin F from nematodas, fishes, amphibians, reptiles, aves, and mammals. Phylogenetic tree showed that the cystatin F from the 20 species is mainly clustered into two groups (Figure 4). One is from the invertebrates, while the other is mainly from the vertebrates (Figure 4). Furthermore, cystatin F in the vertebrate cluster is classified into two groups (Figure 4). One group is from fishes, amphibians, reptiles, aves, and mammals, and the other group is from agnathans (Figure 4). Phylogenetic analysis showed Lm-cystatin F was clustered as the out group of the cystatin F from fishes, amphibians, reptiles, aves, and mammals.

### 2.3. Lm-cystatin F was Expressed as a His-Tag Fusion Protein

**Figure 5:**
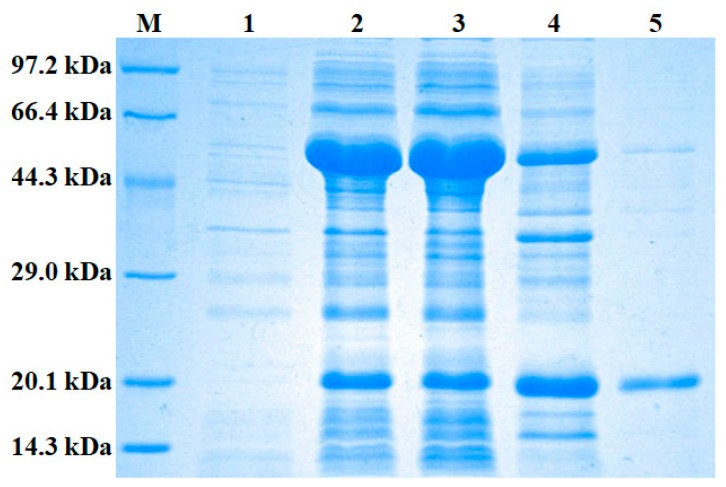
The expressed rLm-cystatin F was detected by 12% SDS-PAGE. After recombination, the rLm-cystatin F was expressed in the presence of pTf16. The expressed rLm-cystatin F was purified through an affinity chromatography column and detected by 12% SDS-PAGE. M, low molecular weight protein standard; 1, the chaperone competent cells were not induced with L-Arabinose (0.5 mg/mL) and IPTG (0.1 mM); 2, the chaperone competent cells were induced with L-Arabinose (0.5 mg/mL) and IPTG (0.1 mM) at 15 °C for 24 h; 3, after ultrasonication and centrifugation, the supernatant of the induced cells; 4, after ultrasonication and centrifugation, the precipitate of the induced cells; 5, the purified rLm-cystatin F.

In order to further reveal the functions of cystatin F in *L. morii*, Lm-cystatin F was recombinant and expressed in the present study. After cloning the optimized sequence into a pCold I vector, Lm-cystatin F was expressed with the induction of 0.5 mg/mL L-Arabinos and 0.1 mM isopropyl-β-d-thiogalactoside (IPTG). As shown in Figure 5, the recombinant protein was detected in both the supernatant and precipitate of the chaperone competent cells. Additionally, the recombinant protein was further identified as a recombinant Lm-cystatin F (rLm-cystatin F) through the analysis from matrix-assisted laser desorption/ ionization time of flight (MALDI-TOF/TOF) mass spectrometry (Figure S2). Due to the His-tag, the rLm-cystatin F was purified through an affinity column and migrated as a single band on 12% sodium dodecyl sulfate-polyacrylamide gel electrophoresis (SDS-PAGE) (Figure 5). Bicinchoninic Acid (BCA) detection showed the concentration of the rLm-cystatin F was 1 mg/mL. Additionally, the residual endotoxin (lipopolysaccharide, LPS) in the 3 and 7 µM rLm-cystatin F was about 0.2 EU/mL (0.08 ng) and 0.42 EU/mL (0.17 ng), respectively. Furthermore, we also obtained a recombinant protein in *Escherichia coli* (*E. coli*) named rLj-26, which is a mutant of rLj-RGD3, and it also migrated as a single protein band on 16.5% Tricine SDS-PAGE [28]. The residual LPS in 7 µM rLj-26 was about 0.35 EU/mL. This protein was used as a control protein to exclude the effects of the His-tag and residual LPS in rLm-cystatin F.

### 2.4. rLm-cystatin F Blocked the Activity of Papain and the Proliferation of HUVECs

**Figure 6:**
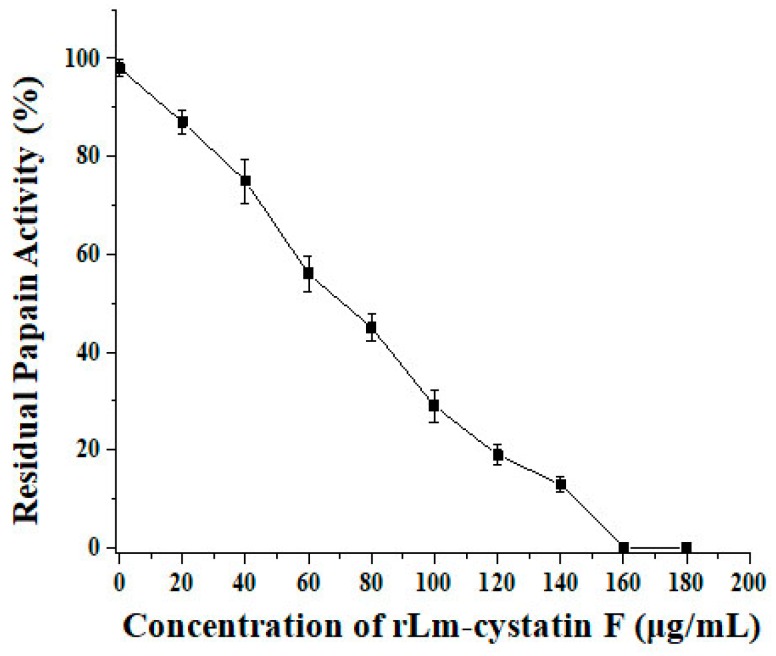
rLm-cystatin F inhibited the activity of papain. Different concentrations of rLm-cystatin F (0, 20, 40, 60, 80, 100, 120, 140, 160, and 180 µg/mL, final concentration) were respectively added into the casein solution in the presence of papain. Residual papain activity was calculated based on the formula mentioned in the methods.

**Figure 7:**
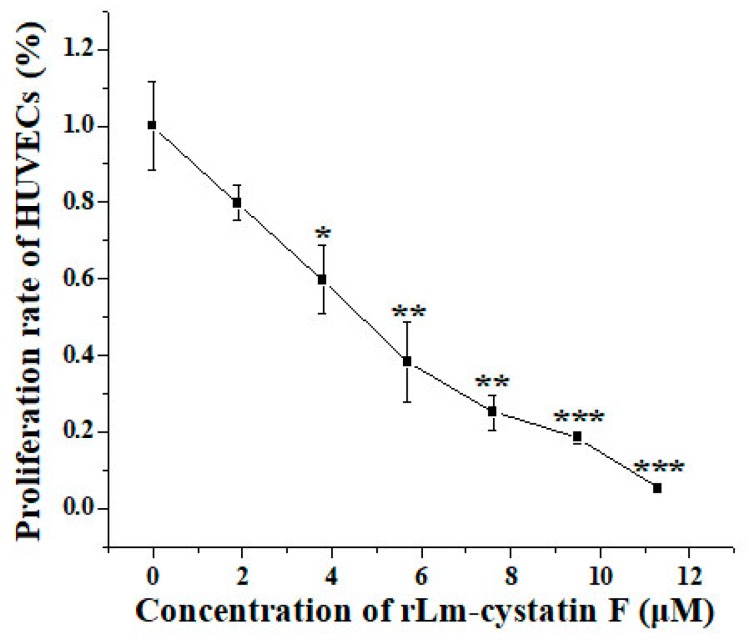
MTT assay showed the inhibitory effects of rLm-cystatin F on the HUVEC’s proliferation. PBS was used as a negative control. The same volume of rLm-cystatin F (0, 1.9, 3.8, 5.7, 7.5, 9.4, and 11.3 μM, final concentration) was added into the HUVECs in the 96-well plates at 37 °C for 24 h. Relative to the PBS group, * *p* < 0.05; ** *p* < 0.01, and *** *p* < 0.001.

In order to detect whether rLm-cystatin F has biological functions, we firstly analyzed the inhibitory effect of rLm-cystatin F on the activity of papain with casein as a substrate. As shown in Figure 6, the degradation of casein catalyzed by papain was inhibited in a dose-dependent manner as the concentration of rLm-cystatin F increased. As the concentration of rLm-cystatin F reached 160 µg/mL, the papain activity was almost not detected. As previously reported, recombinant snake venom cystatin (sv-cystatin) from Taiwan cobra (*Naja naja atra*) showed anti-angiogenic activity [18,29]. Whether Lm-cystatin F is also capable of disturbing the angiogenic process has not been reported yet. In the present study, various concentrations (0, 1.9, 3.8, 5.7, 7.5, 9.4, and 11.3 μM) of rLm-cystatin F were incubated with HUVECs at 37 °C for 24 h. According to our 3-(4,5-dimethylthiazol-2-yl)-2, 5-diphenyltetrazolium bromide (MTT) assay, rLm-cystatin F showed inhibitory effects on the proliferation of HUVECs dose-dependently (Figure 7). The half inhibitory concentration (IC_50_) of rLm-cystatin F on HUVECs’ proliferation was 5 μM (Figure 7). Compared with the phosphate buffered saline (PBS, negative control) group, less than 10% HUEVCs were alive after treating with 11.3 μM rLm-cystatin F (Figure 7). Furthermore, 0.08 and 0.17 ng LPS, which are, respectively, equal to the content of the residual endotoxin in the 3 and 7 μM rLm-cystatin F, did not inhibit the proliferation rate of HUVECs at the same conditions (Figure S3).

### 2.5. rLm-cystatin F Suppressed the Adhesive, Migrated, and Invasive Processes of HUVECs

**Figure 8:**
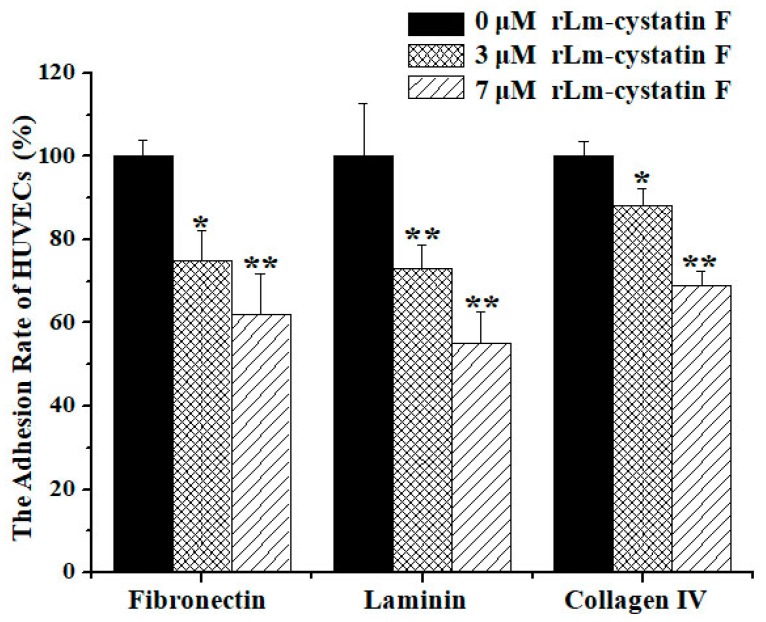
MTT assay showed that rLm-cystatin F thwarted HUVECs adhered to fibronectin, laminin, and collagen IV. PBS (0 µM rLm-cystatin F) was used as a negative control. Relative to the PBS group, * *p* < 0.05 and ** *p* < 0.01.

**Figure 9:**
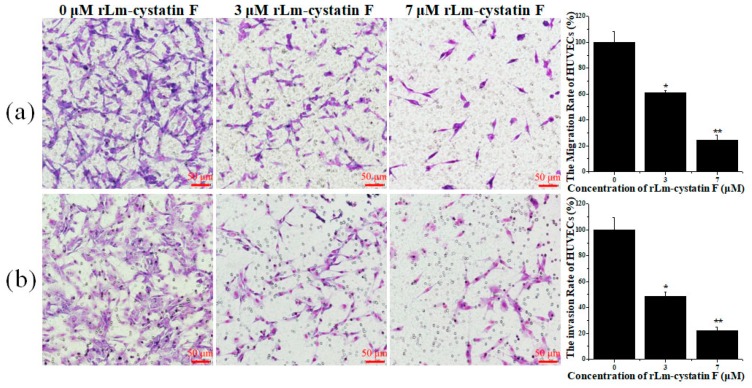
Transwell assays showed the inhibitory effects of rLm-cystatin F on the HUVEC’s migration (**a**) and invasion (**b**). PBS was used as a negative control. Relative to the PBS group, * *p* < 0.05 and ** *p* < 0.01.

Besides properties that caused inhibitory effects on the proliferation of endothelial cells, other anti-angiogenic factors were also reported to affect the adhesive, migrated, and invasive abilities of the endothelial cells [30]. Therefore, we used three extracellular matrix proteins, MTT, and Transwells, the classic assays to detect whether rLm-cystatin F is also able to affect the adhesive, migrated and invasive activities of HUVECs. In the presence of rLm-cystatin F, the HUVECs that were adhered to the three extracellular matrix proteins were thwarted significantly (Figure 8). When fibronectin was used as an adhesive molecule, the inhibitory rates of 3 and 7 µM rLm-cystatin F on HUVECs adhesion were 25 ± 7% (*p* < 0.05) and 38 ± 9.8% (*p* < 0.01), respectively. Similarly, 3 and 7 µM rLm-cystatin F suppressed HUVECs adhered to laminin by 27 ± 5.6% (*p* < 0.01) and 45 ± 7.6% (*p* < 0.01), respectively, and thwarted HUVECs adhered to collagen IV by 12 ± 4.3% (*p* < 0.05) and 31 ± 3.5% (*p* < 0.01), respectively. Furthermore, 0.1 and 0.2 ng LPS did not affect the adhesion rate of HUVECs when collagen IV, fibronectin, and laminin were used as adhesive molecules (Figure S3). Even though the lower chambers of the Transwells were full of RPMI 1640 medium with 15% fetal bovine serum (FBS) in order to stimulate the cell migration or invasion, rLm-cystatin F reduced the number of the migrated and invasive HUVECs on the polycarbonate filter of the Transwells (Figure 9). The inhibitory migration rates were 39.3 ± 1.9% (*p* < 0.05) and 75.6 ± 3.6% (*p* < 0.01) in the 3 and 7 μM rLm-cystatin F treating groups, respectively (Figure 9a); while the inhibitory invasion rates were 51.4 ± 3.2% (*p* < 0.05) and 78.2 ± 2.8% (*p* < 0.01) in the 3 and 7 μM rLm-cystatin F treating groups, respectively (Figure 9b). Furthermore, 0.1 and 0.2 ng LPS did not inhibit the migration and invasion of HUVECs (Figure S4). The above results suggested that rLm-cystatin F decreased the abilities of HUVECs on their adhesion, migration, and invasion in a dose-dependent manner.

### 2.6. rLm-cystatin F Reduced the Abilities of Tube Formation from HUVECs

**Figure 10:**
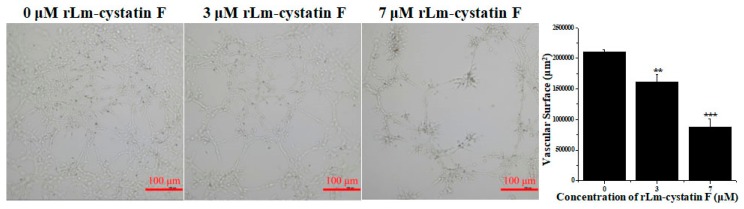
The inhibitory effects of rLm-cystatin F on the tube formation from HUVECs in vitro. PBS was used as a negative control. Relative to the PBS group, ** *p* < 0.01 and *** *p* < 0.001.

In the present study, the anti-angiogenic activity of rLm-cystatin F was analyzed in the classic tube formation assay in vitro. As shown in Figure 10, the HUVECs were able to form tube-like structures on the Matrigel obviously. However, after treating with rLm-cystatin F, the abilities of HUVECs to form the tube-like structures decreased in a dose-dependent manner (Figure 10). The inhibitory rates on the surface of the formed tubes were 23.1 ± 6% (*p* < 0.01) and 58.2 ± 6.23% (*p* < 0.001) in the 3 and 7 μM rLm-cystatin F treating groups, respectively (Figure 10). Furthermore, 0.1 and 0.2 ng LPS did not affect the tube formation of HUVECs in vitro (Figure S5). Similarly, the control protein, rLj-26, which was also expressed in *E. coli* and contained a His-tag as well as the residual LPS, did not inhibit the tube formation of HUVECs in vitro (Figure S6).

## 3. Discussion

In the present study, a cystatin F homologue was cloned from the buccal gland secretion of *L. morii* for the first time. Based on the characterization, cystatins are usually classified into three groups, including stefins (family 1), cystatins (family 2), and kininogens (family 3) [1]. As the amino acid sequence of Lm-cystatin F contains a signal peptide and two disulfide bonds at the carboxyl terminal, which are the classic characterizations of family 2, Lm-cystatin F should be classified into the family 2 in the cystatin superfamily [1]. Although sequence alignments displayed that Lm-cystatin F has relatively lower sequence identity with the cystatin F from the other species, Lm-cystatin F still possesses the highly-conserved motifs, which indicated that Lm-cystatin F might also exert the typical functions of the cystatin superfamily. Furthermore, phylogenetic analysis showed Lm-cystatin F was clustered as the out group of the cystatin F from the other vertebrates. This is also in accordance with the evolutional pattern, as agnathans (*L. morii*) are one of the most primitive vertebrates. Actually, lots of proteins identified from agnathans are clustered as a single group, which is located at the bottom of the phylogenetic tree [31].

To date, previous studies used affinity chromatography, reverse-phase chromatography, and ion-exchange chromatography to purify the native cystatin from the venom of the snakes to reveal its biological functions [29,32]. The shortcomings of this method are that a lot of venom from the snakes is required to be collected and the relatively lower yield of the proteins, as well as the multiple steps. At present, the cystatins are usually expressed in the *Pichia pastoris* (*P. pastoris*) or insect cells probably due to the relatively complex structures, which might affect their expression and folding [13,16]. Actually, cystatins were usually expressed as inclusion bodies in the *E. coli* [33,34]. Although some studies used pEGX-4T-1, pSmart-I, or pET32a vector to obtain the soluble cystatins in the *E. coli*, the recombinant cystatins usually contain a tag with a relatively larger molecular weight that needs to be removed or set in an empty plasmid control to eliminate its effects on the functions of the recombinant cystatins [35,36,37]. At first, we subcloned the Lm-cystatin F into a pCold I vector, however, the expressed rLm-cystatin F was hardly detected in the *E. coli* (our unpublished data). As Lm-cystatin F contains eight rare codons in its cDNA sequence, we speculated that the rare codons might lead to the difficult expression of Lm-cystatin F in *E. coli*. Thus, we optimized the sequence of Lm-cystatin F to replace the rare codons without changing its amino acid sequence. With the help of a plasmid named pTf16, which would help the recombinant protein form correct folding, rLm-cystatin F was obtained as a soluble protein with only a His-tag. Importantly, the yield of rLm-cystatin F was also improved and it showed inhibitory effects on papain activity and angiogenesis. This method helped us acquire the rLm-cystatin F in a relatively simple step, which provided novel information for the expression of the other members in the cystatin superfamily in the *E. coli* system. Actually, the expression of recombinant proteins in *E. coli* usually encounters aggregation or degradation due to their inability to form correct folding [38]. At present, the synergetic expression of trigger factor (TF) with the target proteins would help solve this problem [38]. In the future, we would also try to use virus or eukaryotic systems to express Lm-cystatin F and compare its activity with rLm-cystatin F expressed in *E. coli* to check whether rLm-cystatin F formed the same folding as that expressed in virus or eukaryotic systems.

In the present study, the His-tag in rLm-cystatin F was not removed. According to the previous studies, a recombinant protein named rLj-GRIM19, which was identified in the buccal glands of *Lampetra japonica*, was expressed in *E. coli* with a His-tag (unpublished data). Additionally, rLj-GRIM19 was proved not to be able to inhibit novel blood vessel generation in chorioallantoic membrane (CAM) models, suggesting that rLj-GRIM19 did not possess anti-angiogenic activity [39]. In our present study, a control protein (rLj-26), which was also expressed in *E. coli* and contained a His-tag, was also proved not to suppress tube formation of HUVECs in vitro. This further indicated that the His-tag would not affect the functions of rLm-cystatin F. Furthermore, the residual imidazole in the purified rLm-cystatin F was removed through ultrafiltration as the molecular weight of imidazole is only 68 Da, which is far smaller than that of rLm-cystatin F (19.85 kDa). During the expression in *E. coli*, LPS might be accompanied with the production of recombinant proteins. Additionally, it needs to be removed in extremely special conditions. In the present study, we did not remove the residual LPS in the purified rLm-cystatin F, as LPS was reported to promote angiogenesis, which was contrary to the functions of rLm-cystatin F [40,41]. Furthermore, the residual LPS in rLm-cystatin F did not inhibit the proliferation, adhesion, migration, invasion, and tube formation of HUVECs. In addition, the residual LPS was also not removed from the purified rLj-26, and rLj-26 did not possess anti-angiogenic activity. This further confirmed that the residual LPS did not affect the functions of rLm-cystatin F. In the future, we would remove residual LPS to meet the criterion of genetic engineering drugs.

According to the previous studies, cystatins participate in a variety of physiological activities and are closely related to the initiation of several diseases in abnormal conditions [1,2,3,4,5,6,7,8,9,10]. Besides, lots of studies also focused on the cystatins in the venom glands of snakes, as well as the cystatins in the salivary glands and the midgut of bloodsuckers. In snakes, cystatins have been extensively identified from a variety of snake species, including the African puff adder (*Bitis arietans*), Japanese Habu (*Trimeresurus flavoviridis*), Taiwan cobra (*Naja naja atra*), pit viper (*Bothrops jararaca*), Brown Treesnake (*Boiga irregularis*), elapid, etc. [21,29,32,42,43,44]. Additionally, some of the cystatins have been reported to inhibit the activity of cysteine proteases in the papain family [29]. In the present study, rLm-cystatin F was found to inhibit the activity of papain, which is a classic cysteine protease (EC 3.4.22.2), suggesting that rLm-cystatin F possessed the basic functions of cystatins [45]. At the similar conditions, rLm-cystatin F showed a better inhibitory effect on the activity of papain when compared with rEsCystatin, which was a recombinant protein identified from the Chinese mitten crab (*Eriocheir sinensis*) [34]. In 2011, Xie and colleagues showed that sv-cystatin from *Naja naja atra* was able to suppress the growth, invasion, and metastasis of B16F10 cells and MHCC97H cells by reducing the activity of cathepsin B, matrix metalloproteinase-2 (MMP-2), and matrix metalloproteinase-9 (MMP-9), and inhibition of epithelial-mesenchymal transition [16,17]. Two years later, the same authors put forward that sv-cystatin was also able to suppress tumor angiogenesis by reducing the level of vascular endothelial growth factor (VEGF)-A165, basic fibroblast growth factor (bFGF), and fms-related tyrosine kinase 1 (Flt-1) [18]. The authors concluded that family 2 cystatins, such as sv-cystatin, might target papain-type cysteine proteases and MMPs to suppress angiogenesis [18]. This is coincident with our observations, as cystatin F from the buccal glands of *L. morii* was also able to repress angiogenesis based on our in vitro studies. Although Lm-cystatin F shares 29% identity with sv-cystatin, it also suppressed crucial steps of angiogenesis, including the proliferation, adhesion, migration, invasion, and tube formation of endothelial cells (HUVECs), probably due to the highly-conserved motifs in its amino acid sequence. In addition, previous studies have reported that papain and some cysteine proteases could promote angiogenesis [46,47]. This suggests that rLm-cystatin F might inhibit angiogenesis by interaction with papain or other cysteine proteases probably due to its inhibitory effects on papain activity. As a member of family 2 cystatins, rLm-cystatin F might also affect the activity of MMPs to block angiogenesis. During the bloodsucking period, the anti-angiogenic activity of Lm-cystatin F might help *L. morii* inhibit the wound healing process of the host fishes. However, further studies are still required to clarify the detailed mechanisms of Lm-cystatin F on anti-angiogenesis. In addition, previous studies have shown that angiogenesis is closely related to tumor progression as novel blood vessels would provide nutrition for the tumor cells, thus, rLm-cystatin F might also affect the activity of tumors [30]. However, further studies of the effects of rLm-cystatin F on tumor cells are still required in the near future.

## 4. Materials and Methods

### 4.1. Cloning of a Cystatin F Homologue (Lm-cystatin F) from the Buccal Glands of L. morii

The handling of live animals was approved by the Animal Welfare and Research Ethics Committee of the Institute of Dalian Medical University (Permit Number: SYXK2004—0029). In the present study, the *L. morii* were captured in December of 2015 in Yalu River in Liaoning province of China. After collecting the buccal glands of *L. morii* in an RNase-free tube, total RNA was immediately extracted through a TakaRa MiniBEST Universal RNA extraction Kit (TaKaRa, Dalian, China). According to the instructions of the manufacturer, the total RNA was used to synthesize cDNA templates by a PrimeScript^TM^ RT-PCR Kit (TaKaRa, Dalian, China). Bases on the ORF sequence (Accession number: ENSPMAP00000007215) in the Ensembl database (www.ensembl.org), the primers for Lm-cystatin F, were designed and listed as follows: 5’-ATGTCCCGTGTGGCATCGTTGTC-3’; 5’-TTAGGCGTTTGGCATGGTAGAAGGT-3’. After PCR amplifications, Lm-cystatin F was subcloned into a pMD^®^ 19-T Vector and sequenced by a PRISMTM 3730XL DNA Analyzer (ABI, Carlsbad, CA, USA).

### 4.2. Sequence Analysis, Alignment, and Phylogenetic Tree Construction

The amino acid sequence and bioinformatic analysis of Lm-cystatin F were performed on the website listed in Table S1. Except Lm-cystatin F, additional 19 cystatin F sequences were obtained from the nematodas, fishes, amphibians, reptiles, aves, and mammals on ExPASy (http://www.expasy.ch/tools/blast). The multiple sequence alignments of cystatin F were performed by ClustalX 1.83 software. A neighbor-joining tree was constructed by MEGA 4.0 software based on the pair-wise deletion of gaps/missing data and a p-distance matrix of an amino acid model with 1000 bootstrapped replicates.

### 4.3. Expression, Purification, and Identification of rLm-cystatin F

Firstly, the sequence of Lm-cystatin F was synthesized by substituting the rare codons in the original sequence of Lm-cystatin F (TaKaRa, Dalian, China). Then, the primers with *EcoR* I and *Pst* I restriction sites were designed based on the optimized sequence of Lm-cystatin F and listed as follows: 5’-GGAATTCCTGCCGGAAACCCGTTG-3’; 5’-AACTGCAGTTAAGCGTTCGGCATGG TAG-3’. Subsequently, the optimized sequence of Lm-cystatin F was subcloned into a pCold I vector and transformed into chaperone competent cells with a plasmid named pTf16 (Chaperone Competent Cell BL21 Series Kit, TaRaKa, Dalian, China) at the same time. After induction with 0.5 mg/mL L-Arabinose and 0.1 mM IPTG at 15 °C for 24 h, the cells were collected through centrifugation at 4 °C and washed with 10 mM Tris-HCl buffer containing 25 mM NaCl and 10 mM imidazole. The rLm-cystatin F was purified through a HisTrap affinity column (GE, Boston, MA, USA) equilibrated with the above Tris-HCl buffer and eluted with the 40–400 mM imidazole in a gradient concentration. Next, 30 mL rLm-cystatin F was firstly concentrated to 2 mL rLm-cystatin F through Amicon^®^ Ultra-15 10K Centrifugal Filter Devices (Millipore, Billerica, MA, USA). Then, 10 mL PBS was added into the above filter and further ultrafiltrated to 2 mL. This step was repeated for three times. After ultrafiltration, the concentration and the purity of rLm-cystatin F were, respectively, detected by a BCA Protein Assay kit (Thermo SCIENTIFIC, Waltham, MA, USA) and 12% SDS-PAGE. The protein band of rLm-cystatin F on 12% SDS-PAGE was digested in-gel by trypsin (25 mM, Promega, Madison, WI, USA) and analyzed by MALDI-TOF/TOF mass spectrometry (Bruker, Billerica, MA, USA). Furthermore, a control protein, rLj-26, was obtained based on the methods reported in the previous study [28]. The residual endotoxin in rLm-cystatin F and rLj-26 was, respectively, detected according to the instructions of the manufacturer (ToxinSensor^TM^ Chromogenic LAL Endotoxin Assay Kit, Nanjing, China).

### 4.4. Enzyme Activity Assay

Based on the previous studies, the inhibitory effect of rLm-cystatin F on the activity of cysteine proteases was analyzed by using casein as a substrate [34]. Briefly, casein (5 mg/mL, final concentration) was firstly dissolved in 50 mM Tris-HCl buffer (pH 7.6) in the presence of 2 mM cysteine-HCl and 0.1 mM EDTA, and then incubated at 37 °C for 10 min. Subsequently, papain (0.1 mg/mL, final concentration) and rLm-cystatin F with various concentrations (0, 20, 40, 60, 80, 100, 120, 140, 160, and 180 µg/mL, final concentration) were, respectively, added into the above reactions and further incubated at 37 °C for 10 min. After mixing with coomassie brilliant blue G250 for 20 min at room temperature, the absorbance at 595 nm was detected. The residual papain activity was calculated based on the following formula:

[1 − (OD595 − OD595’)/OD595’] × 100%(1)

OD595 indicated the absorbance of the reactions which contained casein, papain, and rLm-cystatin F; while OD595’ indicated the absorbance of the reaction, which contained only casein and papain. Triplicate experiments were performed independently.

### 4.5. HUVECs’ Culture and MTT Assay

HUVECs were obtained from Dr. Jihong Wang in Liaoning Normal University. HUVECs were cultured in the medium named RPMI 1640 (GIBCO, Grand Island, NY, USA) in the presence of 10% FBS (GIBCO, Grand Island, NY, USA) in a CO_2_ incubator (Thermo, Waltham, MA, USA). After trypsin digestion, the HUVECs were put into 96-well plates and cultured in the incubator for 24 h. rLm-cystatin F was diluted to different concentrations from 0–11.3 μM with the above medium without FBS. The same volumes of PBS and rLm-cystatin F were incubated with the HUVECs in the incubator for 24 h. Then, 5 mg/mL MTT solution was put into the above HUVECs for 4 h. After removing the medium without drawing the formazan, 100 μL dimethyl sulfoxide (DMSO) was added into the HUVECs and incubated at 37 °C for 10 min. The absorbance at 492 nm of the HUVECs in the presence of PBS and rLm-cystatin F was recorded by a microplate reader (Thermo SCIENTIFIC, Waltham, MA, USA). The proliferative rate of HUVECs was calculated according to our previous studies [30]. At the same conditions, the effects of LPS on the proliferation rate of HUVECs were also detected by MTT assay.

### 4.6. Adhesion, Migration, and Invasion Assays

After digestion, the HUVECs were collected and resuspended with the FBS-free 1640 medium. PBS, 3, and 7 μM rLm-cystatin F were then added into the HUVECs. During the adhesive assays, fibronectin, laminin, as well as collagen IV, were dissolved in PBS buffer with final concentration of 0.1 mg/mL and then were, respectively, used to coat the 96-well plates at low temperatures. After removal of the extracellular matrix proteins, the pretreated HUVECs were put into the above 96-well plates and incubated in the CO_2_ incubator for 2 h (collagen IV) or 3 h (fibronectin and laminin). Subsequently, the HUVECs without adhesion were washed with PBS buffer and the adhesive HUVECs were measured with MTT assay. During the migration assays, the pretreated HUVECs were put into the upper chamber of the Transwells. Meanwhile, the 1640 medium with 15% FBS was added into the lower chamber of the Transwells. Twenty hours later, the polycarbonate filters were fixed with the fixative. After removal from the Transwells, the filters were put onto the slides and stained with the Wright-Giemsa solution according to the previous studies [48]. Four views were randomly selected and captured with an inverted fluorescence microscope (Nikon, Tokyo, Japan). The migrated HUVECs were counted with the NIS-Elements D software and analyzed according to the method reported by Qi Jiang and colleagues [30]. In addition, the first step in the invasion assays was to add 4 mg/mL Matrigel (BD Bioscience, New York, NY, USA) in the upper chamber of the Transwells. After incubation at 37 °C for 30 min, the upper and lower chambers of the Transwells were, respectively, added with the PBS or rLm-cystatin F (3 and 7 μM) treated HUVECs and 1640 medium with 15% FBS. The subsequent procedures were similar to the migration assays. The time for invasion assays was 36 h. At the same conditions, the effects of LPS on the adhesion, migration, and invasion rate of HUVECs were also detected by MTT and Transwell assays.

### 4.7. Anti-Angiogenic Activity Assay

Firstly, both the 96-well plates and tips were put at 4 °C overnight. Subsequently, the Matrigel (4 mg/mL, final concentration) was diluted with the 1640 medium in the absence of FBS and then added into the 96-well plates. After incubation at 37 °C for 40 min, the PBS, 3, and 7 μM rLm-cystatin F were, respectively, mixed with the HUVECs and then added into the above 96-well plates. After 10 h, the inverted fluorescence microscope was used to observe the tube formation from the HUVECs. Four views were randomly selected and captured. The total area of the tubes was analyzed by the NIS-Elements D software. At the same conditions, the effects of LPS and rLj-26 on the tube formation ability of HUVECs were also observed with PBS as a control according to our previous studies [30].

### 4.8. Statistical Analysis

The experiments were performed three times, each in triplicate. Student’s *t*-test was used to analyze the differences between the PBS and rLm-cystatin F treating groups. A statistical significance was shown as followed: * *p* < 0.05; ** *p* < 0.01, and *** *p* < 0.001.

## 5. Conclusions

This is the first report to show the characterization of a cystatin F homologue (Lm-cystatin F) identified from the buccal glands of *L. morri*. After cloning and recombination, Lm-cystatin F was successfully expressed as a soluble protein in the *E. coli* system with a His-tag. Although Lm-cystatin F shares low sequence identity with the members from the cystatin superfamily, rLm-cystatin F still inhibited papain activity and showed anti-angiogenic activity, which suggested that Lm-cystatin F might be an important protein to suppress the wound healing process of host fishes and might be used as a potential anti-angiogenic drug in the future.

## Figures and Tables

**Table 1:** The sequence identity of Lm-cystatin F with the other cystatin F.

Category	Amino Acids	Species	Accession Number	Identity (%)
Mammals	145	*Homo sapiens*	CAD52872.1	33
Mammals	144	*Mus musculus*	NP_034107.2	32
Mammals	145	*Hipposideros armiger*	XP_019488972	31
Aves	147	*Gallus gallus*	NP_001186323	32
Aves	147	*Columba livia*	PKK20598.1	31
Aves	147	*Buceros rhinoceros silvestris*	XP_010137948.1	30
Amphibians	149	*Xenopus laevis*	NP_001091281	32
Amphibians	145	*Alligator mississippiensis*	KYO18358.1	38
Amphibians	145	*Chrysemys picta bellii*	XP_005293586	35
Amphibians	145	*Pelodiscus sinensis*	XP_006136954	31
Reptiles	145	*Eublepharis macularius*	JAC94872	32
Reptiles	154	*Python regius*	JAC94922	31
Fishes	143	*Oncorhynchus mykiss*	XP_021420522.1	35
Fishes	143	*Salmo salar*	NP_001134364.1	34
Fishes	133	*Esox lucius*	NP_001297968.1	38
Fishes	128	*Danio rerio*	NP_001082882	36
Fishes	140	*Takifugu rubripes*	XP_011601516	32
Agnathans	152	*Lampetra morii*	MG902948	100
Nematodas	136	*Necator americanus*	ETN77353.1	27
Nematodas	141	*Ancylostoma duodenale*	KIH58790.1	26

## Supplementary Materials

The following are available online at http://www.mdpi.com/1660-3397/16/12/477/s1, Figure S1: The identification of a cystatin F homologue from the buccal glands of feeding *L. morii*. Figure S2: The expressed rLm-cystatin F was identified by mass spectrometry. Figure S3: LPS did not inhibit the proliferation and adhesion rate of HUVECs. Relative to the PBS group, * *p* < 0.05. Figure S4: LPS did not inhibit the migration and invasion rate of HUVECs. Relative to the PBS group, * *p* < 0.05. Figure S5: LPS did not affect the tube formation of HUVECs in vitro. Figure S6: rLj-26 did not inhibit tube formation of HUVECs in vitro. Table S1: The websites and softwares were used to analyze the amino acid sequence and bioinformatic information of Lm-cystatin F.
