# Supplementary material for: The Anti-Angiogenic Activity of a Cystatin F Homologue from the Buccal Glands of Lampetra morii
Source: Mar Drugs. 2018 Nov 29;16(12):477. doi: 10.3390/md16120477 (PMC6316161; doi:10.3390/md16120477)
Supplement: Supplementary file 1 [file marinedrugs-16-00477-s001.pdf]

marine drugs

MDPI

Supplementary Material

**Accession:**	ENSPMAP00000007215	**Score:**	73.41
**Database:**	lamprey	**MW [kDa]:**	17.10
**Seq. Coverage [%]:**	11.10 %	**pI:**	10.31
		**No. of Peptides:**	2

```
        10         20         30         40         50         60         70         80         90        100        110        120
MSRVASLSLL LCGLYCCCCC AALPETRWRP MLGAPTAIQP TDPGLHTAAA EATRRFNSGL NSRTVYRLDR VTKATRQIVS GLKYIFEADL KSTECLKSEE RVAEDCNFHD DGVATVFKCR
       130        140        150        160
FEVWTIPWRK QTKVLSQSCQ QNNPIKPSTM PNA
```

Cmpd.	No. of Cmpds.	m/z meas.	Δ m/z [ppm]	z	Rt [min]	Score	P	Range	Sequence	Modification
1168	2	499.7650	3.48	2	33.4	30.6	0	84-91	K.YIFEADLK.S	
2203	3	617.3259	2.48	2	41.6	42.9	0	121-129	R.FEVWTIPWR.K	

BRUKER

Detailed Protein Report

Project Info

**Name:**	libowen	**Date:**	April 16, 2014

Sample Info & Protocols

		**Date:**	January 26, 2015
**Name:**	20150125B5		

Search Result Info

Search Result	Location	Search Engine	Database	Ident. Compounds
Impact_PH_130411_Mascot_2015-01-26 06:17:31	/libowen/20150125B5/ProteinAnalysisResults.mgf	Mascot, 2.4.0	lamprey, lamprey_20130124.fasta	109/3589

Figure S1. The identification of a cystatin F homologue from the buccal glands of feeding *L. morii*.

Project Info

**Name:**	NANO-U3000	**Date:**	April 17, 2018

Sample Info & Protocols

		**Date:**	July 5, 2018
**Name:**	201807152S		

Search Result Info

Search Result	Location	Search Engine	Database	Ident. Compounds
Impact_PH_130411_Mascot_2018-07-19 06:24:16	/NANO-U3000/201807152S/20180712B8_RB8_01_2183.mgf	Mascot, 2.4.0	LIP, LIP_01.fasta	7/5635

**Protein 1:**	>CystatinF		
**Accession:**	CystatinF	**Score:**	231.98
**Database:**	LIP	**MW [kDa]:**	17.10
**Seq. Coverage [%]:**	46.70 %	**pI:**	9.77
		**No. of Peptides:**	6
**Modification(s):**	Carboxymethyl		

Cmpd.	No. of Cmpds.	m/z meas.	Δ m/z [ppm]	z	Rt [min]	Score	P	Range	Sequence	Modification
2113	4	504.2650	-4.19	2	24.9	46.6	0	54-62	R.LFNSGLNSR.T	
2653	3	862.4787	-7.37	2	29.8	59.3	1	76-90	R.QIVSGLKYIFEADLK.S	
2319	1	962.9243	1.62	2	26.8	18.4	0	101-117	R.VAEDCNFHDDGVATVFK.C	Carboxymethyl: 5
2916	13	617.3160	-13.62	2	32.2	46.8	0	120-128	R.FEVWTIPWR.K	
2633	5	681.3698	-3.09	2	29.6	32.3	1	120-129	R.FEVWTIPWRK.Q	
2223	7	1108.5174	-2.14	2	25.9	28.6	0	133-152	K.VLSQSCEQNNPIKPSTMPNA.-	Carboxymethyl: 6

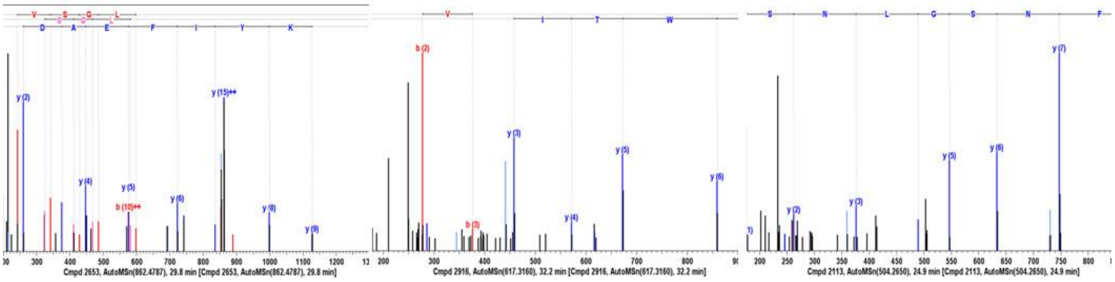

Figure S2. The expressed rLm-cystatin F was identified by mass spectrometry.

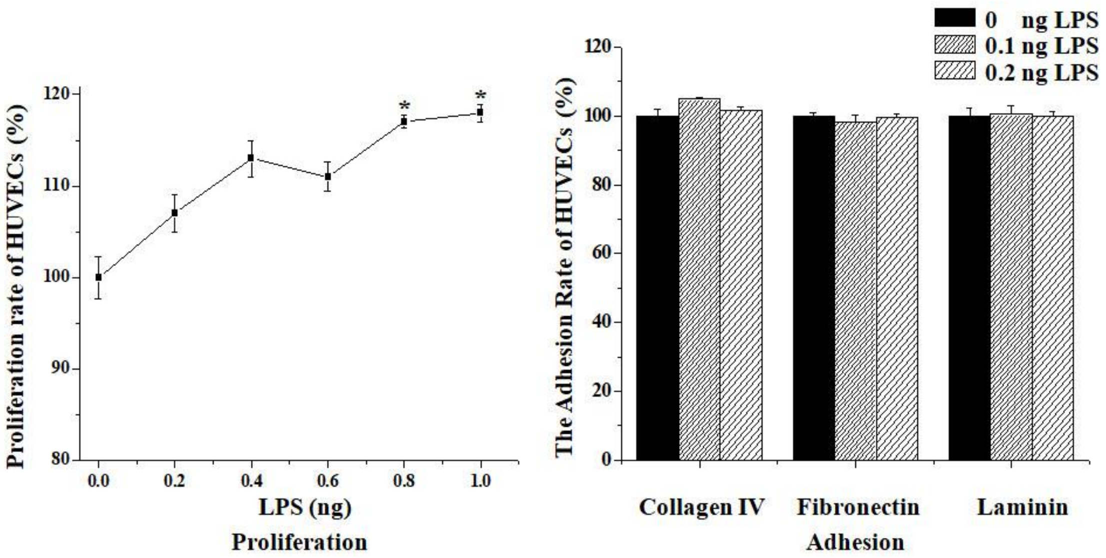

Figure S3. LPS did not inhibit the proliferation and adhesion rate of HUVECs. Relative to the PBS group, * $p < 0.05$.

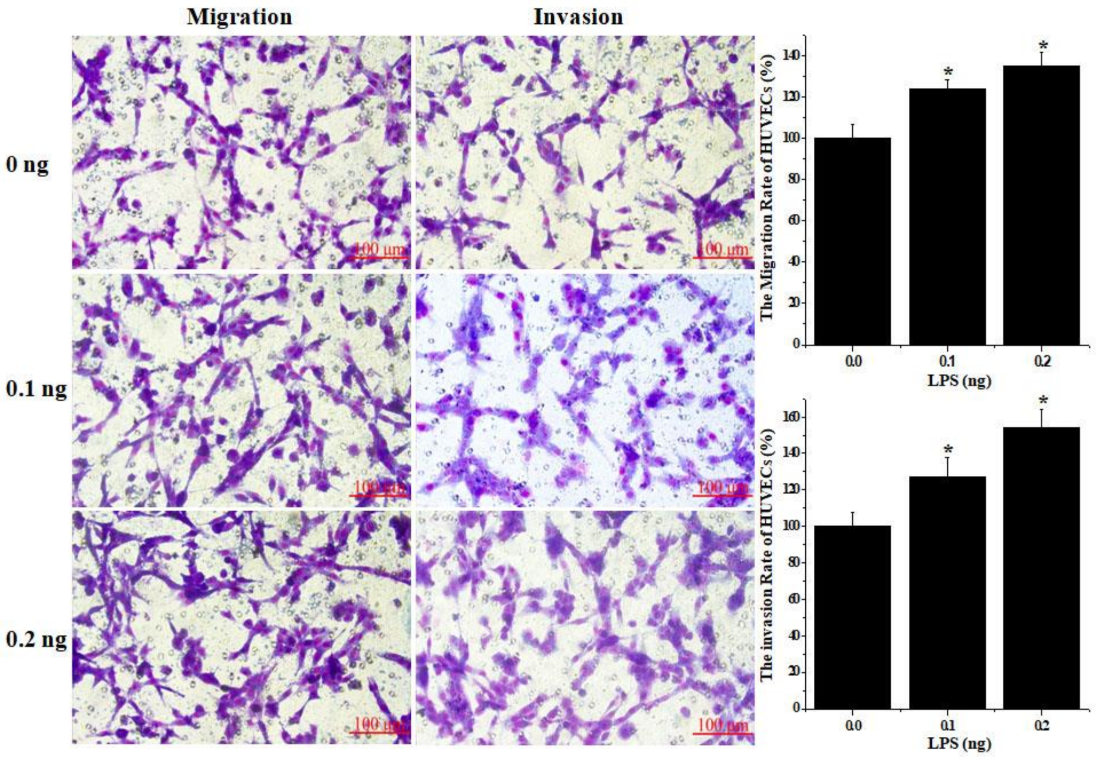

Figure S4. LPS did not inhibit the migration and invasion rate of HUVECs. Relative to the PBS group, * $p < 0.05$.

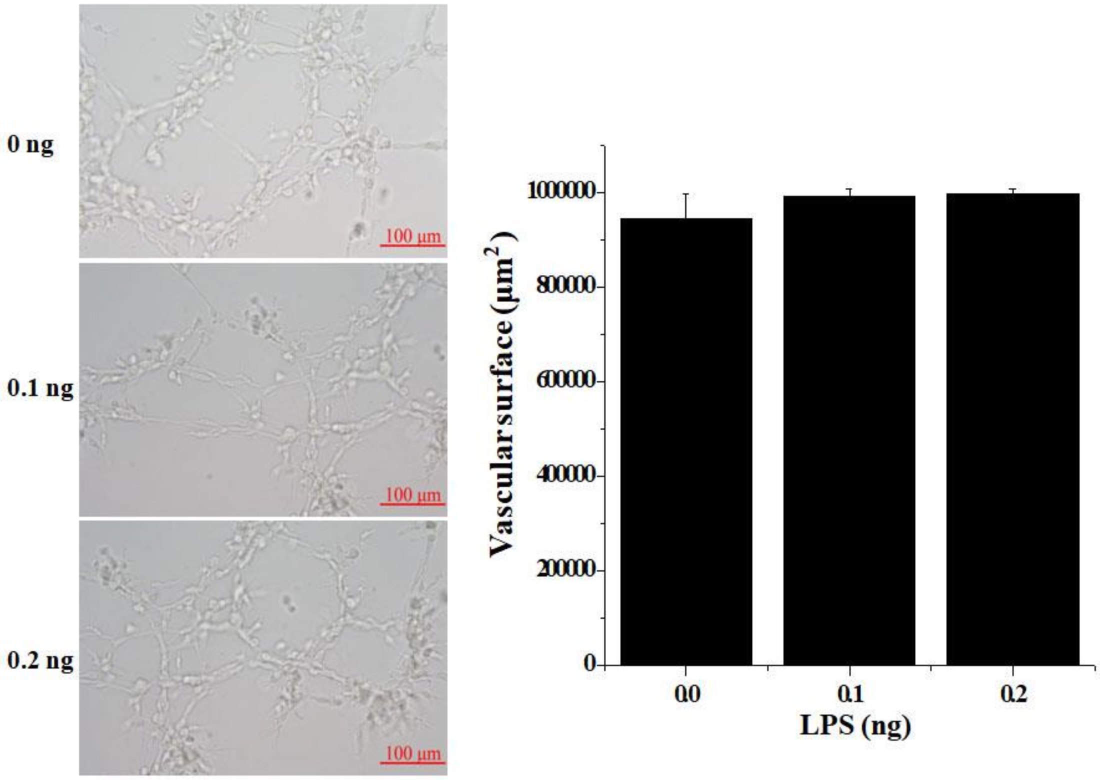

Figure S5. LPS did not affect the tube formation of HUVECs *in vitro*.

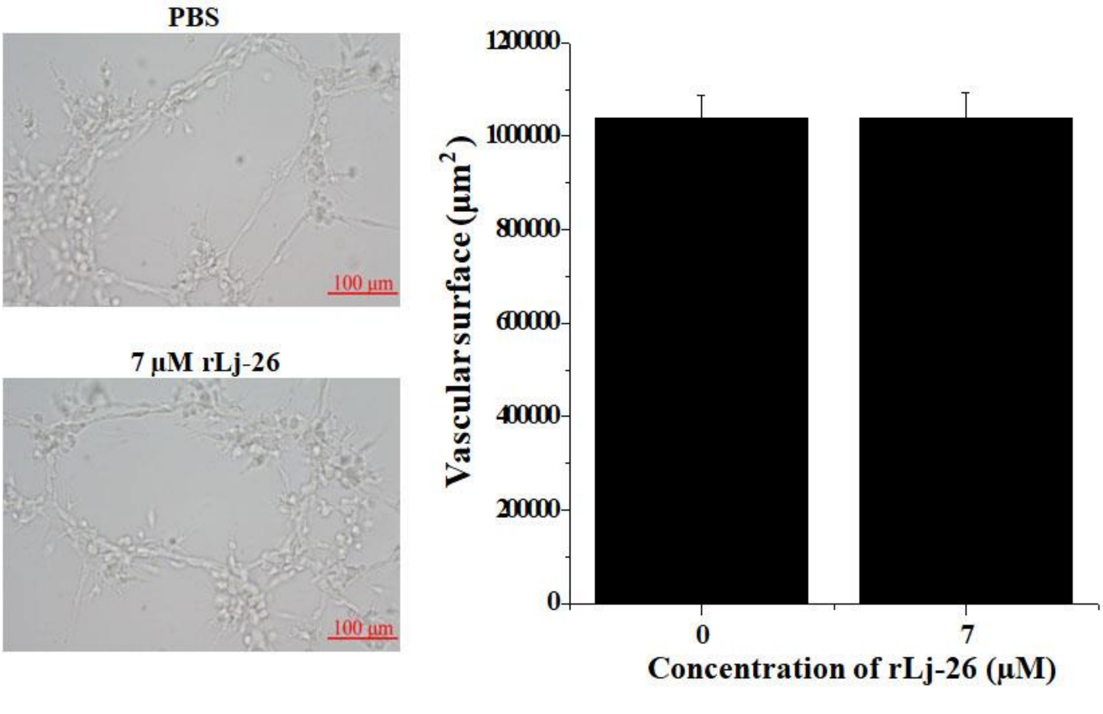

Figure S6. rLj-26 did not inhibit tube formation of HUVECs *in vitro*.

Table S1. The websites and softwares were used to analyze the amino acid sequence and bioinformatic information of Lm-cystatin F.

Predictions	Websites and softwares
ORF	http://www.ncbi.nlm.nih.gov/gorf/gorf.html
Physicochemical property	http://web.expasy.org/protparam/
Signal peptide	http://www.cbs.dtu.dk/services/SignalP/
Functional domains	http://smart.embl-heidelberg.de/
Three dimensional structure	http://swissmodel.expasy.org and PyMOL software
